# The RhoA/ROCK Pathway Ameliorates Adhesion and Inflammatory Infiltration Induced by AGEs in Glomerular Endothelial Cells

**DOI:** 10.1038/srep39727

**Published:** 2017-01-05

**Authors:** Jialing Rao, Zengchun Ye, Hua Tang, Cheng Wang, Hui Peng, Weiyan Lai, Yin Li, Wanbing Huang, Tanqi Lou

**Affiliations:** 1 Division of Nephrology, Department of Medicine, The Third Affiliated Hospital of Sun Yet-sen University, Guangzhou, Guangdong 510630, China

## Abstract

A recent study demonstrated that advanced glycation end products (AGEs) play a role in monocyte infiltration in mesangial areas in diabetic nephropathy. The Ras homolog gene family, member A Rho kinase (RhoA/ROCK) pathway plays a role in regulating cell migration. We hypothesized that the RhoA/ROCK pathway affects adhesion and inflammation in endothelial cells induced by AGEs. Rat glomerular endothelial cells (rGECs) were cultured with AGEs (80 μg/ml) *in vitro*. The ROCK inhibitor Y27632 (10 nmol/l) and ROCK1-siRNA were used to inhibit ROCK. We investigated levels of the intercellular adhesion molecule 1 (ICAM-1) and monocyte chemoattractant protein1 (MCP-1) in rGECs. *Db/db* mice were used as a diabetes model and received Fasudil (10 mg/kg/d, n = 6) via intraperitoneal injection for 12 weeks. We found that AGEs increased the expression of ICAM-1 and MCP-1 in rGECs, and the RhoA/ROCK pathway inhibitor Y27632 depressed the release of adhesion molecules. Moreover, blocking the RhoA/ROCK pathway ameliorated macrophage transfer to the endothelium. Reduced expression of adhesion molecules and amelioration of inflammatory cell infiltration in the glomerulus were observed in *db/db* mice treated with Fasudil. The RhoA/ROCK pathway plays a role in adhesion molecule expression and inflammatory cell infiltration in glomerular endothelial cells induced by AGEs.

Diabetic nephropathy (DN) is one of the most serious and common complications leading to end-stage renal disease (ESRD) several years after diabetes onset^1^. The exact mechanisms of DN are not yet clear. In recent years, DN was thought to be affected by disorders of glucose metabolism, change in hemodynamics, cytokines and genetic background^2^,^3^.

Diabetes is characterized by chronic hyperglycemia and the development of diabetes-specific microvascular pathology^3^ involving advanced glycation end products (AGEs), resulting from hyperglycemia-elicited metabolic and hemodynamic derangements, which have been proven to contribute to vascular complications in diabetes^4^. Some *in vitro* studies have shown that AGEs induce vascular damage though oxidative stress in diabetes^5^, while the interaction between the glomerular endothelium and AGEs is unknown.

One major group of small GTPases, the Rho GTPases (average molecular weight 20–40 kDa), regulate the cell junction, cell cytoskeleton and cell migration^6^. RhoA is the most recognized member of the Rho GTPase family. ROCK, which exists in two isoforms, ROCK1 and ROCK2, is a downstream effector of RhoA. It was reported that ROCK is critical in controlling migration, proliferation, cell apoptosis/survival, gene transcription and differentiation^7^. However, the role of the RhoA/ROCK pathway in the regulation of adhesion and inflammation in the glomerulus in DN has not yet been clarified. This study examined ROCK1 as it is primarily distributed in the kidneys.

Inflammation plays a key role in the onset and development of DN. Human biopsies and animal models have indicated the presence of macrophages in diabetic kidneys^8^. Adhesion and migration of macrophages to the endothelium are divided into four steps: chemotaxis, adhesion, transformation and shuttling. These biological behaviors require adhesion molecules and chemokines. The endothelium plays a critical role in adhesion and migration. The relationship between macrophages and glomerular endothelial cells (GECs) is worthy of discussion.

The intercellular adhesion molecule-1 (ICAM-1) and the chemokine monocyte chemoattractant protein-1 (MCP-1) have a significant effect on cell adhesion, proliferation and inflammatory cell infiltration^9^. ICAM-1 precedes the transendothelial migration of inflammatory cells from the capillary bed into tissues^10^. It is known that MCP-1 rapidly causes rolling monocytes to adhere firmly onto monolayers and it plays a role in monocyte recruitment^11^. In the present study, ICAM-1 and MCP-1 were assessed to determine the influence of AGEs on adhesion and the inflammatory infiltration of GECs. The role of RhoA/ROCK signaling in *db/db* mice was also determined to evaluate the mechanism of inflammatory cell infiltration in DN.

In conclusion, we attempted to determine the role of the RhoA/ROCK pathway in adhesion and inflammatory infiltration induced by AGEs, and assessed whether the RhoA/ROCK pathway plays a role in the progression of DN.

## Results

### AGEs activate RhoA/Rho-kinase in rat glomerular endothelial cells (rGECs)

**Figure 1:**
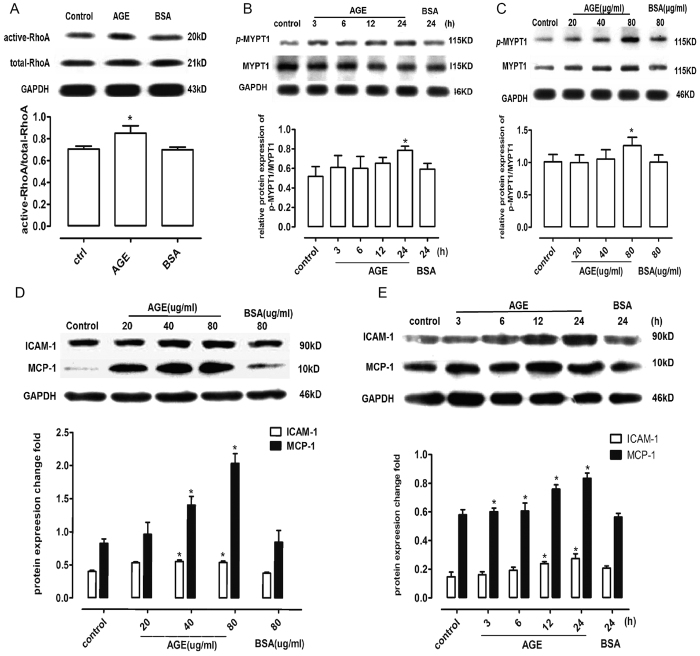
AGEs activate RhoA/ROCK activity and stimulate the secretion of ICAM-1 and MCP-1 in rGECs. (**A**) Active RhoA detected by the RhoA pull down assay. (**B**) Protein expression of *p*-MYPT1, MYPT1 and GAPDH at various times. (**C**) Protein expression of *p*-MYPT1, MYPT1 and GAPDH at different AGE doses. (**D**) Protein expression of ICAM-1 and MCP-1 induced by AGEs at incremental doses. (**E**) Protein expression of ICAM-1 and MCP-1 induced by AGEs (80 μg/ml) at various time points. *P < *0.05 vs. control, n* = *6.*

The RhoA pull down assay and ROCK activity assay were performed as described in the methods. RhoA was activated by AGEs after 3 hours of rGEC stimulation (Fig. 1A). Increases in *p*-MYPT1 expression were stimulated by both incremental doses of AGEs or a single 80 μg/ml dose (Fig. 1B,C).

### AGEs stimulate the secretion of ICAM-1 and MCP-1

RGECs were incubated with gradually increasing doses of AGEs for 24 hours. ICAM-1 and MCP-1 were expressed after incubation (Fig. 1D). The secretion of adhesion molecules and chemokines increased as the incubation time increased (Fig. 1E).

### AGE-mediated enhancement of adhesion molecule and chemokine secretion in rGECs requires RhoA/ROCK signaling

**Figure 2:**
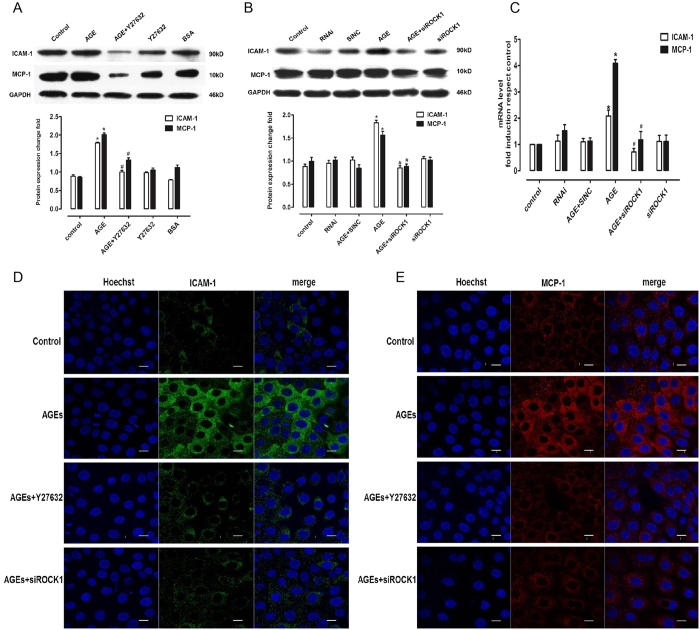
Blocking the RhoA/ROCK pathway inhibits AGE-induced enhancement of adhesion molecules and chemokines. (**A**) Protein expression of ICAM-1 and MCP-1 in GECs incubated with Y27632. (**B**) Protein expression of ICAM-1 and MCP-1 in GECs transfected with ROCK1 siRNA. (**C**) mRNA expression of ICAM-1 and MCP-1 in GECs transfected with ROCK1 siRNA. (**D**) Immunofluorescence of ICAM-1 with different doses. (**E**) Immunofluorescence of MCP-1 with different doses. Bar = 5 μm, *P < *0.05 vs. control*, ^*#*^*P* < *0.05 vs. AGEs, n* = *6.*

Y27632 treatment for 30 minutes significantly inhibited AGE-induced ICAM-1 and MCP-1 upregulation (Fig. 2A). Further studies showed that silencing the expression of ROCK1 by ROCK1 siRNA transfection blocked AGE-induced upregulated secretion of adhesion molecules and chemokines (Fig. 2B,C). Immunofluorescence staining was then performed to evaluate the expression of adhesion molecules and chemokines in endothelial cells incubated with AGEs. The results showed that the expression levels of adhesion molecules and chemokines in cells incubated with AGEs were higher than in cells co-incubated with AGEs and the Y27632 ROCK inhibitor. We also found that the expression of ICAM-1 and MCP-1 was significantly inhibited in cells transfected with siROCK1 (Fig. 2D,E).

### AGE-induced migration of macrophages to endothelial cells requires RhoA/ROCK signaling

**Figure 3:**
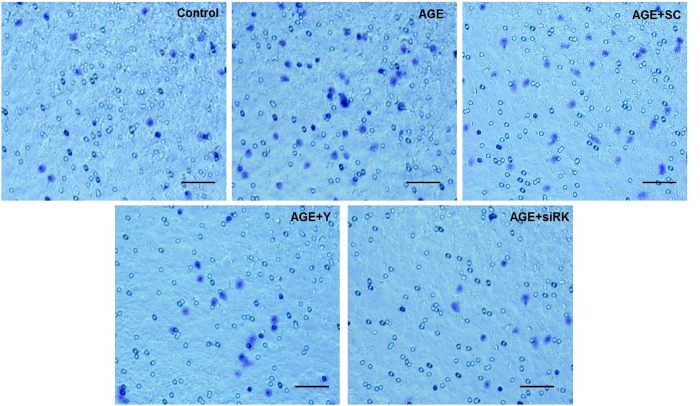
AGE-induced macrophage migration to endothelial cells requires the RhoA/ROCK pathway. Macrophages in the gel with crystal violet. Bar = 100 μm. *P < *0.05 vs. control*, ^*#*^*P* < *0.05 vs. AGEs, n* = *6.* siRK, siROCK1. SC, scramble RNA, Y, Y27632.

RGECs were cultured in the lower chambers of the 12-well transwell plate, and pretreated with Y27632 (10 nmol/l) or transfected with ROCK1 siRNA. The medium was replenished and incubated with AGEs. Peritoneal macrophages were added to the upper chambers. After the cells were incubated for 6 hours and stained with 0.1% crystal violet, the number of macrophages on the lower surface of the membrane was then counted under a microscope. The results showed that treatment with AGEs significantly increased cell migration when compared to the control group. On the other hand, down-regulation of ROCK1 activity with the Rho kinase inhibitor Y27632 or ROCK1-siRNA rescued AGE-induced migration of macrophages. There were no significant changes in the negative control group (Fig. 3).

### Fasudil affects the physiology and pathology in db/db mice

**Figure 4:**
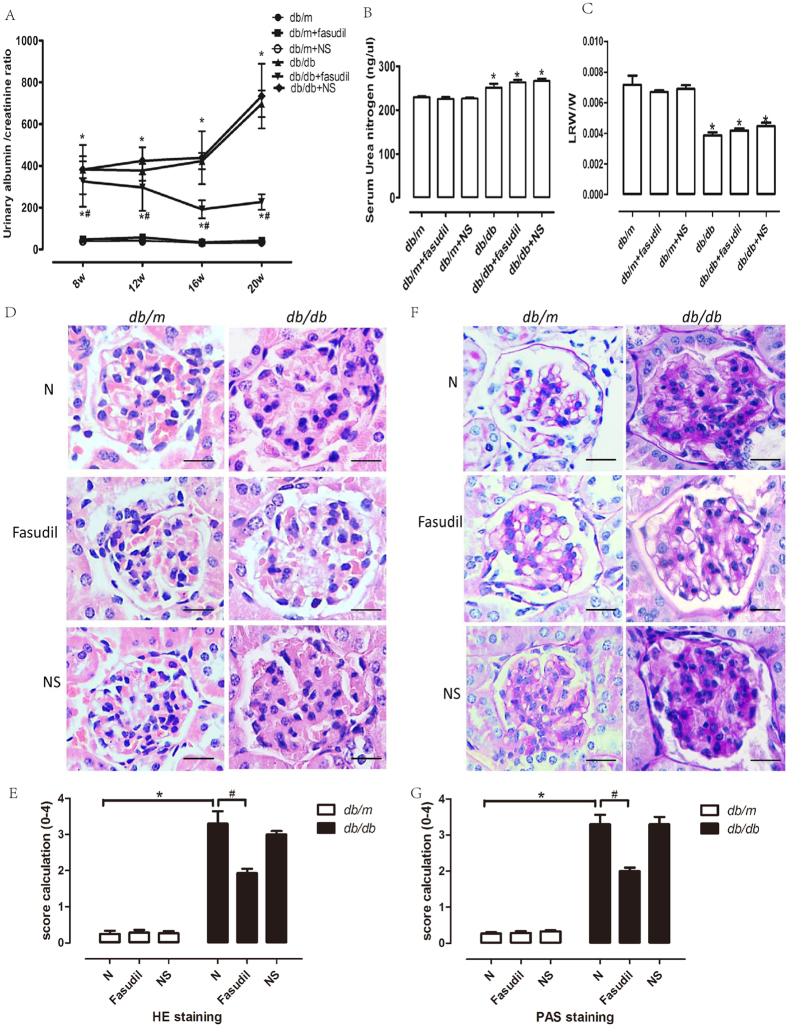
Fasudil ameliorated pathological injury in glomeruli of DN *db/db* mice. (**A**) Urinary albumin to creatinine ratio. (**B**) Serum urea nitrogen. (**C**) Left kidney weight to body weight ratio. (**D**) HE staining. Bar = 20 μm. (**E**) Score calculation of HE staining. (**F**) PAS staining. Bar = 20 μm. (**G**) Score calculation of PAS staining. *P < *0.05 vs. db/m mice*^*#*^*P* < *0.05 vs. db/db mice, n* = *6.* LRW, left renal weight. W, weight.

To eliminate the effects of normal saline, which was used to dilute the Fasudil freeze-dried powder, we divided the *db/m* and *db/db* mice into three groups: the normal control group (N group), the Fasudil group and the normal saline group (NS group). Body weight, blood glucose levels and urine volumes of the *db/db* mice were measured and recorded (Table 1). Obvious symptoms of diabetes, such as weight gain, increased appetite, water intake, urine and lethargy were observed in the *db/db* mice at approximately 8–10 weeks. As expected, the mean blood glucose levels in *db/db* mice were notably higher than in *db/m* mice. Nevertheless, Fasudil did not affect the blood glucose in diabetic mice (Table 1). The urinary albumin-to-creatinine ratio (UACR) was significantly higher in *db/db* mice when compared to *db/m* mice at the age of 8 weeks or older. After 12 weeks of treatment with Fasudil, *db/db* mice exhibited marked decreases in the UACR levels (Fig. 4A). Serum urea nitrogen in *db/db* mice was also higher than in *db/m* mice, which revealed that *db/db* mice had developed diabetic renal injury. Treatment with Fasudil had no effect on serum urea nitrogen (Fig. 4B). The ratio of kidney weight to body weight was also determined. The results showed that treatment with Fasudil for 12 weeks did not alter the kidney weight to body weight ratio in db/db mice (Fig. 4C). As shown by hematoxylin eosin (HE) staining, distinct cell hyperplasia and infiltration of lymphocytes and macrophages were observed in the glomeruli of *db/db* or normal-saline-injected *db/db* mice, by contrast, the matrix was ameliorated in the *db/db* mice treated with Fasudil, and there was no difference between the untreated group and the normal saline treatment group (Fig. 4D). Figure 4E provides a graphical representation of the HE score calculation for each group. The HE scores increased from 0.25 ± 0.08 in *db/m* mice to 3.3 ± 0.34 in *db/db* mice as a result of diabetic nephropathy. Treatment with Fasudil decreased the scores significantly from 3.3 ± 0.34 in the disease control group to 1.93 ± 0.11. The representative glomerular histology of periodic acid-Schiff (PAS) staining is shown in Fig. 4E. Compared to the control *db/m* mice, the glomerular accumulation of the PAS-positive matrix was prominent in *db/db* mice or saline-treated *db/db* mice. The matrix was lower in Fasudil-treated *db/db* mice (Fig. 4F). As shown in Fig. 4G, the PAS score increased from 0.27 ± 0.04 in *db/m* mice to 3.3 ± 0.26 in *db/db* mice. Treatment with Fasudil significantly decreased the score from 3.3 ± 0.26 in disease control group to 2.0 ± 0.1.

### Fasudil inhibits RhoA/ROCK activity in mice with diabetic nephropathy

**Figure 5:**
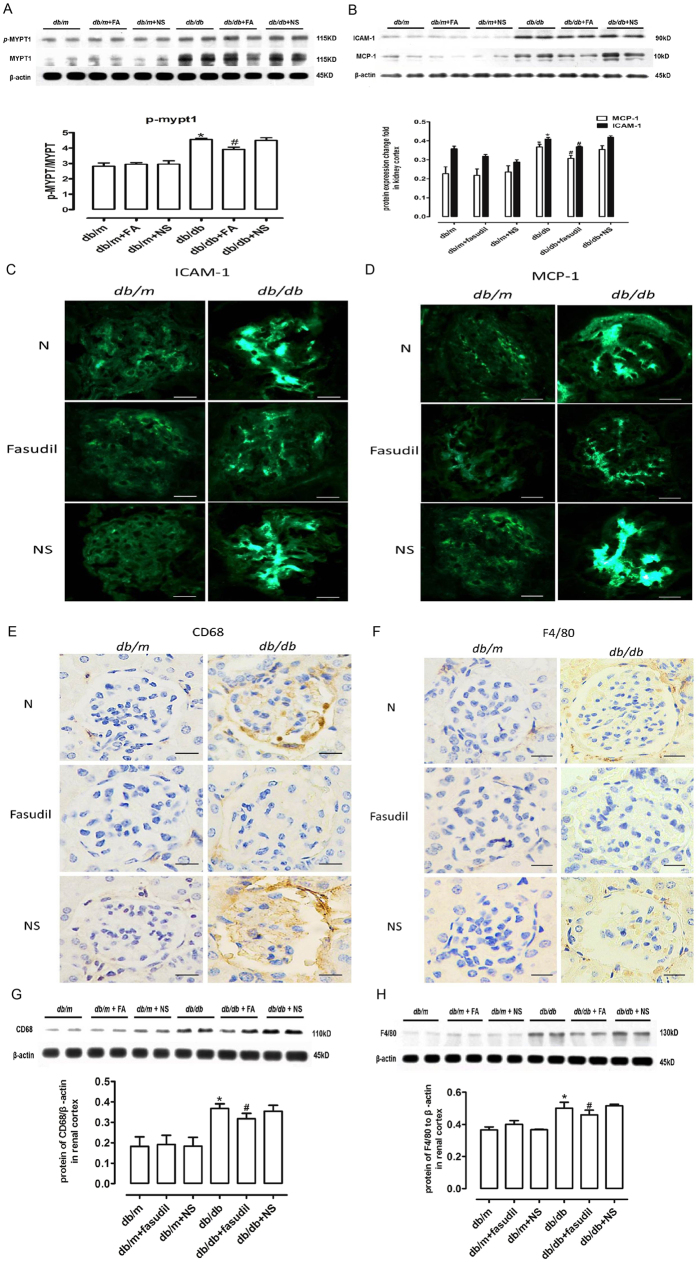
Fasudil ameliorated inflammatory infiltration in DN *db/db* mice. (**A**) Western blot analysis of *p*-MYPT1, MYPT1 and β-actin. (**B**) Western blot analysis of ICAM-1 and MCP-1. (**C**) Immunofluorescence of ICAM-1 in glomeruli. Bar = 20 μm. (**D**) Immunofluorescence of MCP-1 in glomeruli. Bar = 20 μm. (**E**) Immunohistochemistry of CD68. Bar = 20 μm. (**F**) Western blot analysis of CD68 and β-actin. (**G**) Immunohistochemistry of F4/80. Bar = 20 μm. (**H**) Western blot analysis of CD68 and β-actin. *P < *0.05 vs. db/m mice*, ^*#*^*P* < *0.05 vs. db/db mice, n* = *6.*

ROCK activity in the kidney cortex was directly assessed with MYPT1, a downstream target of ROCK. As shown in Fig. 5A, upregulation of *p*-MYPT1 in *db/db* mice indicated increases in ROCK activity. However, the *db/m* and Fasudil-treated *db/db* mice did not differ. When Fasudil was injected intraperitoneally, the phosphorylation of MYPT1 was suppressed in the kidneys when compared to the *db/db* mice without treatment.

### Fasudil has a significant effect on the expression of adhesion molecules and chemokines in mice with diabetic nephropathy

The protein levels of renal cortex ICAM-1 and MCP-1 in *db/db* diabetic group increased relative to the *db/m* mice or *db/m* mice treated with normal saline. (Fig. 5B), the protein levels of ICAM-1 and MCP-1 in the *db/db* mice treated with Fasudil were significantly reduced when compared to the *db/db* mice. These findings demonstrated higher adhesion activity and inflammation potential in DN. Nevertheless, Fasudil significantly suppressed the activity of the ROCK pathway during diabetic renal injury, revealing that Fasudil had a protective effect on adhesion and inflammation in diabetic kidney disease. Using immunofluorescence staining, we found that Fasudil significantly inhibited the expression of ICAM-1 and MCP-1 in glomerular endothelial cells (Fig. 5C,D).

### Fasudil ameliorates macrophage infiltration in db/db mice

Immunohistological staining of CD68 and F4/80 was used to locate the macrophages. There was no visible staining in the *db/m* mice or negative controls. By contrast, the CD68 and F4/80 levels increased in the glomeruli of *db/db* mice and *db/db* mice treated with normal saline, indicating that glomerular inflammation is present in DN. There was no staining in the *db/db* mice treated with Fasudil (Fig. 5E,F). In addition, the protein levels of CD68 and F4/80 in the renal cortex increased in *db/db* mice and *db/db* mice treated with normal saline. After Fasudil treatment, the protein levels of CD68 and F4/80 were significantly inhibited when compared to the diabetic mice (Fig. 5G,H).

## Discussion

In the present study, AGEs induced the secretion of adhesion molecules and chemokines *in vitro*, and inhibited the RhoA-ROCK pathway and thereby ameliorated cell adhesion and inflammatory infiltration. *In vivo*, sequential injections of Fasudil ameliorated cell adhesion and inflammation in DN glomeruli, reduced urinary protein and protected kidney function.

Research on ECs in relation to the pathomechanisms of DN has included oxidative stress and fibrosis. Inflammation and the infiltration of inflammatory cells in the glomerulus also have been observed in DN. Our study indicated that AGEs significantly stimulated the secretion of adhesion molecules and chemokines in glomerular endothelial cells (GECs). We further demonstrated that inhibition of RhoA/ROCK signaling ameliorated the release of adhesion molecules and inflammation around the glomerulus in *db/db* mice with DN, indicating that the Rho/ROCK pathway plays a role in glomerular inflammation in DN.

The pathobiochemistry of AGEs could explain many of the changes observed in diabetes-related complications^12^. Important work has been carried out in the last two decades to elaborate the chemical processes and pathways initiated by AGEs, however, these processes and pathways have yet to be fully clarified. In a study by Yuji *et al*., AGEs upregulated the levels of VCAM-1 mRNA in human umbilical vein endothelial cells (HUVECs)^13^. Yamagishi *et al*.^14^ showed that AGEs stimulated MCP-1 expression in mesangial cells and were associated with monocyte infiltration in mesangial areas during the early phase of DN. We attempted to determine how AGEs upregulated the expression of adhesion molecules and chemokines in GECs. Once combined with receptors on the GEC membrane, AGEs can activate a series of effectors downstream. Activation of the AGE receptor (RAGE) bound to RhoA has been demonstrated. Hirose *et al*. found that AGEs activate RhoA. Activated RhoA forms a complex with RAGE on HUVECs resulting in cell contraction with actin reorganization and transendothelial hyperpermeability which can be inhibited by Y27632 and anti-RAGE antibodies^15^. Using RAGE−/− mice injected with streptozotocin (STZ) to establish diabetes models, Ishibashi *et al*.^16^ showed that RAGE deficiency was uniquely renoprotective in normalizing albuminuria due to its ability to restore the diabetes-induced decrease in renal ATP production. Although high glucose is involved in the pathogenesis of diabetes, there is a growing body of evidence to indicate that AGEs play an important role in diabetic vascular inflammatory complications. Our study demonstrated that AGEs influence the secretion of ICAM-1 and MCP-1 in glomerular ECs. The present study demonstrated that AGEs induced dose-dependent and time-dependent expression of ICAM-1 and MCP-1 in GECs, revealing that AGEs can stimulate adhesion and inflammatory infiltration in GECs. The effect of AGEs on glomerular endothelial cells is one of the factors involved in diabetic glomerular inflammation.

Furthermore, we sought to gain deeper insight into the molecular mechanisms by which AGEs regulate rGEC secretion of adhesion molecules. RhoA/ROCK signaling has recently received a considerable amount of attention because of its renoprotective effects in DN. Inhibition of the RhoA/ROCK pathway can benefit the kidneys in diabetics. Xiaoyan Wu *et al*.^17^ found that caspase activation amplifies tumor necrosis factor-α (TNF) -induced inflammation in renal endothelial cells. In cultured renal ECs, TNF induced apoptosis through a caspase-8-dependent pathway. TNF caused the translocation of the p65 subunit of NF-κB to the nucleus, resulting in the upregulation of inflammatory markers, such as the adhesion molecules ICAM-1 and VCAM-1. They also found that TNF induced rapid but transient activation of RhoA. TNF-induced caspase was activated upstream of RhoA activation. RhoA/ROC kinase drives multiple complex changes in the actin of the cytoskeleton.

The RhoA/ROCK pathway has also attracted considerable amounts of attention in various fields of research, especially in the cardiovascular field, and plays an important role in various cellular functions involved in the pathogenesis of cardiovascular disease and the effects of many vasoactive substances^18^. In a study by Li *et al*.^19^, the ROCK inhibitor, Fasudil, attenuated the high glucose-induced increase in VCAM-1 and MCP-1 expression in HUVECs, revealing that inhibition of the Rho/ROCK pathway has the potential to protect against the diabetic inflammatory process in vessels. Thus, we hypothesized that AGEs induced the secretion of inflammatory markers in GECs through the activation of the RhoA/ROCK pathway. However, there were many types of Rho kinase inhibitors. As shown by Xi *et al*.^20^, the Chinese drug, berberine, blocked RhoA/ROCK signaling and inhibited increases in glucose-induced expression of ICAM-1, TGF-β and fibronectin (FN) in glomerular mesangial cells, demonstrating that RhoA/ROCK pathway-associated adhesion also took place in the glomerulus. *In vivo* studies of the RhoA/ROCK pathway have also been conducted. Peng *et al*. demonstrated that the Rho kinase inhibitor Fasudil reduced collagen IV produced by mesangial cells and decreased cortical TGF-β in the glomerulus of STZ-induced diabetes in SD rats, indicating that the RhoA/ROCK pathway plays a role in fibrosis^21^. Y27632, a relatively specific Rho kinase inhibitor was used in our *in vitro* experiments. We designed ROCK1 siRNA that specifically inhibit ROCK1. Fasudil was used in the *in vivo* experiment; however, it inhibits the Rho kinase ubiquitously. We demonstrated that AGEs induce RhoA and ROCK activity. This activation was depressed by Y27632 and ROCK1 siRNA. The secretion of ICAM-1 and MCP-1 induced by AGEs were also decreased by Rho kinase inhibitors, verifying that the RhoA/ROCK pathway contributes to the expression of adhesion molecules and chemokines relative to the inflammatory activity.

The pathogenesis of DN is multifactorial. An appropriate animal model that restored the pathophysiological status was chosen in the present study. The *db/db* mutation on the C57BLKS background was used in our study. This mutation has been intensively investigated and exhibits many features similar to human DN^22^. In our study, models of DN were established with a distinct clinical presentation, such as high fasting blood glucose, increased blood urea nitrogen and urine albumin-creatinine ratio. Inflammation is thought to be one of the most important aspects in the pathogenesis of DN. A study had demonstrated that expressions of ICAM-1 and MCP-1 in aortic tissues were increased significantly in *db/db* mice compared with C57BL/6 J mice. In addition, resveratrol, a natural antioxidant and anti-inflammatory substance, was found to ameliorate the expression of inflammatory molecules and infiltration of macrophages through inhibiting the activity of NF-κB pathway in aortic tissues of *db/db* mice^22^. According to our findings, inhibition of the RhoA/ROCK pathway exhibited a partial, but significant effect on relieving glomerular inflammation. We verified the enhancement of ROCK1 activity in the renal cortex of *db/db* mice by measuring *p*-MYPT1 protein expression. We found that the RhoA/ROCK pathway inhibitor, Fasudil, partially ameliorated the accumulation of adhesion and infiltration of inflammatory cells in the glomerulus, indicating that the inhibition of the RhoA/ROCK pathway had a protective effect on glomerular inflammation. Our findings provide a new perspective for detecting inflammation in DN.

Human biopsies and animal models have demonstrated the presence of macrophages in diabetic kidneys^23^. We observed that more macrophages adhered to GECs when incubated with AGEs. Blocking the RhoA/ROCK pathway significantly reduced adhesion *in vitro*. These findings revealed that the RhoA/ROCK pathway plays a role in macrophage migration to the endothelium. Fasudil protected the kidneys without inflammation by releasing molecules and chemokines, which attracted inflammatory cells. Xiao *et al*. injected STZ-induced DN rats with calcitriol for 4 months and examined the M2 phenotype in the treatment group. They found that vitamin D prevents podocyte injury via the regulation of the macrophage M1/M2 phenotype in rats with DN^24^. The mechanism of this phenomenon remains unclear, and we would like to detect the phenotype conversion of macrophages in glomeruli with diabetic nephropathy in future studies.

In conclusion, our results showed that AGEs enhanced ICAM-1 and MCP-1 via the RhoA/ROCK pathway in glomerular endothelial cells. In the animal study, inhibition of the RhoA/ROCK pathway ameliorated albuminuria, improved the urinary albumin-creatinine ratio and improved adhesion and inflammatory cell infiltration in the glomerulus. Thus, it is obvious the RhoA/ROCK pathway plays an important role in diabetic nephropathy. Blocking this pathway can protect the kidneys without causing inflammation and improves kidney function. Our findings provide new insights into the mechanisms of diabetic nephropathy and provide a foundation for therapeutic strategies.

## Materials and methods

### Cell experiments

Rat glomerular endothelial cells were kindly provided by Dr. Farhad R. Danesh (MD Anderson Cancer Center, USA). The RGECs were grown in Roswell Park Memorial Institute (RPMI) 1640 medium (GIBCO, Auckland, New Zealand) containing 10% fetal bovine serum (FBS, GIBCO, Australia), 10% cell supplement (BD Biosciences, San Jose, CA, USA), 100 U/ml penicillin (HyClone, Logan, UT, USA) and 100 μg/ml streptomycin (HyClone, Logan, UT, USA) at 37 °C under 5% CO2 in a cell incubator. Four to ten generations of these cells were used in this study. When the cells were 80–90% confluent, we substituted serum-free medium and the cells were rendered quiescent for 24 hours. In the following experiments, the cells were first cultured to 80% confluence. The growth was arrested with RPMI-1640 culture medium without FBS for ~18–24 hours.

The RGECs were cultured with AGE-BSA (Merck Millipore, Billerica, MA, USA) at 20 μg/ml, 40 μg/ml and 80 μg/ml for 3, 6, 12 and 24 hours, respectively. The cells cultured without AGE-BSA served as controls. The rGECs were pretreated with 10 nmol/l Y27632 (Sigma-Aldrich, St. Louis, MO, USA), a specific inhibitor of Rho-kinase^25^, to inhibit the RhoA/ROCK pathway.

### Animal studies

Female *db/db* and *db/m* mice (n = 36, 4 weeks old, Nanjing University, Nanjing, China) were maintained on standard chow and allowed free access to tap water. All animal procedures conformed to the Guide for Care and Use of Laboratory Animals by the National Institute of Health for Laboratory Animal Research and were approved by the Sun Yat-sen University Animal Care and Use Committee. *Db/db* and *db/m* mice were randomly divided into groups and received the following treatments at 8 weeks: Fasudil-injected group (10 mg/kg/day Fasudil by intraperitoneal injection, Chase Sun, Tianjin, China) (n = 6), saline-injected group (0.2 ml saline by intraperitoneal injection) (n = 6), and the normal control group without treatment (n = 6). *Db/m* mice were also divided into corresponding groups. The mice were sacrificed ethically after 12 weeks. Blood samples and kidney cortex tissue were collected and stored −80 °C.

Peritoneal macrophages were extracted from 200–220 g male Sprague-Dawley (SD) rats (Sun Yet-sen University, Guangzhou, China) of either gender. These rats were sacrificed via cervical dislocation after diethyl-ether anesthesia, then dipped into 75% ethyl alcohol for 5 minutes for sterilization, injected intraperitoneally with 10 ml of serum-free Dulbecco’s modified Eagle’s medium (DMEMGIBCO, Auckland, New Zealand) and subsequently removed with a Pasteur pipette. Cells were centrifuged at 1000 rpm for 10 minutes and resuspended in DMEM medium containing 10% FBS, 100 U/ml penicillin and 100 μg/ml streptomycin, and cultured in an adaptive vessel at 37 °C under 5% CO2 in an incubator. The medium was replaced after 2 hours^26^.

### Animal biochemical parameter analysis

The mice were housed in metabolic cages (Nalgene Nunc International, Rochester, NY, USA). Urine was collected over 24 hours and placed into tubes containing antibiotics. Microalbumin was determined with Albuwell M (Exocell, Philadelphia, PA, USA). The values were normalized to creatinine, which was measured using the Creatinine Companion (Exocell, Philadelphia, PA, USA). Fasting blood glucose was measured using the OneTouch UltraSmart Blood Glucose Meter (Lifescan, Milpitas, CA, USA) every 2 weeks. Blood urine nitrogen levels were measured using a blood urea nitrogen enzymatic kit (Bio Scientific, Austin, TX, USA).

### Protein extraction and western blotting

Cell or kidney cortex proteins were extracted by adding radio immunoprecipitation assay (RIPA) lysis buffer (Beyotime Tech, Shanghai, China) containing 1 mM PMSF at 4 °C. The suspension was centrifuged at 14,000 × g and the medium containing the cellular proteins was collected. For western blotting, 10% sodium dodecyl sulfate-polyacrylamide gels (SDS PAGE) were run under standard conditions with 45 μg of protein in each lane. The gels were placed in transfer buffer for 15 minutes and transferred to polyvinylidene fluoride membranes at 100 V for 1 hour. The membranes were rinsed in Tris-buffered saline and blocking buffer (5% milk powder) for 5 minutes, then immersed in blocking buffer for 1 hour before incubation with the following primary antibodies: rabbit monoclonal antibodies against MCP-1 (Abcam, Cambridge, MA, USA) and GAPDH (Protein Tech, Chicago, IL, USA), and mouse monoclonal antibodies against ICAM-1 (Abcam, Cambridge, MA, USA). After rinsing in wash buffer, horseradish peroxidase-conjugated secondary antibody was added for 1 hour. After the final washing, the membranes were developed using enhanced chemiluminescence (ECL, Mougene, China). The examination was repeated three times.

The kidney cortex proteins were extracted as described previously. The protein antibodies used included rabbit monoclonal antibodies against MCP-1 (Cell Signaling Technology, Danvers, MA, USA), β-actin (Cell Signaling Technology, Danvers, MA, USA), rat monoclonal antibodies against F4/80 (Abcam, Cambridge, MA, USA), ICAM-1 (Abcam, Cambridge, MA, USA) and monoclonal antibodies against CD68 (Abcam, Cambridge, MA, USA).

### SiRNA transfection

The sequences of ROCK1 siRNA were as follows: sense 5′-AATACCTTTCTGGGAGTTCT-3′ and antisense 5′-TGAGCATGTCTTTAATCTAC-3′. ROCK1 and the negative control of siRNA transfection were carried out using diluted moderate Lipofectamine RNAiMAX Reagent (Invitrogen, Carlsbad, CA, USA) and ROCK1 siRNA in Opti-MEM medium (GIBCO, Carlsbad, CA, USA) in 1:1 proportions according to the manufacturer’s instructions. The lysates were obtained for the western blot analysis as previously mentioned.

### RhoA pull down assay

RhoA activity was assessed using the pull-down RhoA Activation Assay Kit according to the manufacturer’s instructions (Merck Millipore, Billerica, MA, USA). The cells were washed in cold TBS twice and lysed in ice-cold MLB buffer. The lysates were collected into microfuge tubes and incubated for 60 minutes at 4 °C with 100 μL of Rhotekin agarose to precipitate the GTP-bound RhoA. The agarose beads were resuspended in 25 μL of 2 × Laemmli reducing sample buffer and boiled for 5 minutes. The total lysates were analyzed using western blot and rabbit polyclonal antibody against RhoA.

### ROCK activity assay

ROCK1 phosphorylates the myosin phosphatase target subunit 1 (MYPT1)^27^, known as the myosin-binding subunit (MBS) of myosin light-chain phosphatase, a Thr853. Myosin phosphatase target subunit 1 (*p*-MYPT1) reflects the Rho-kinase activity^28^ as a substrate protein. In brief, cell lysates and kidney cortical tissue extracts were analyzed using western blot analysis and rabbit polyclonal antibodies against *p*-MYPT1 (Santa Cruz, Dallas, TX, USA) and MYPT1 (Abcam, Cambridge, MA, USA). The ratio of *p*-MYPT1 and MYPT1 exhibited ROCK1 activity.

### Migration Assay

A transwell system was used to detect cell migration. The rGECs were cultured in the lower chambers of the 12-well transwell plate. Confluent cells were rendered quiescent via incubation for 24 h in serum-free medium before pretreatment with Y27632 (10 nmol/l) for 30 minutes, or were transfected with ROCK1 siRNA for 6 hours. Then, the medium was renewed and incubated with 80 μg/ml of AGEs for 24 hours. Peritoneal macrophages were added to the upper chambers for 6 hours. The upper compartment was removed and cotton was used to wipe the membrane lightly. The membrane was then fixed with 90% ethanol for 15 minutes, washed three times with distilled water and stained with 0.1% crystal violet. Images were captured using a light microscope.

### Pathological staining

For the morphometric studies, the kidneys were fixed in 10% neutral buffered formalin and subsequently embedded in paraffin. Four-micrometer paraffin sections of kidney cortex tissue were dewaxed. hematoxylin was used to stain the nuclei and eosin was used to dye the cytoplasm. The sections were then dehydrated and sealed with neutral balsam. The 4-μm sections of the paraffin-embedded tissues were stained with periodic acid-Schiff (PAS). Forty consecutive glomerular cross-sections per mouse were analyzed. Digital images of the glomeruli were obtained using light microscopy (400x). The area of the glomeruli and the mesangial matrix was quantified in a blinded fashion using an image analysis system. The mesangial matrix index was calculated as the ratio of the mesangial area to the glomerular area × 100 (% area).

### Immunohistochemistry

Samples of kidney cortex were fixed with 10% neutral-buffered formalin, embedded in paraffin and sliced into 4-μm sections. The sections were then incubated in boiling sodium citrate buffer for antigen retrieval. Specific primary antibodies, anti-F4/80 and anti-CD68 (Abcam, Cambridge, MA, USA), were incubated with the samples overnight. The color was developed using a mixture of 0.05% 3,3′-DAB/0.01% H2O2 for 10 min. The sections were counterstained with Mayer’s hematoxylin for 15 s, dehydrated and mounted for evaluation under light microscopy.

### Statistical analysis

The data are presented as the means ± SD. The between-group comparisons were performed using one-way ANOVA for unbalanced designs, followed by Student’s t-test for paired and unpaired data when appropriate. A *P*-value less than 0.05 was considered statistically significant.

## Additional Information

**How to cite this article:** Rao, J. *et al*. The RhoA/ROCK Pathway Ameliorates Adhesion and Inflammatory Infiltration Induced by AGEs in Glomerular Endothelial Cells. *Sci. Rep.* **7**, 39727; doi: 10.1038/srep39727 (2017).

**Publisher's note:** Springer Nature remains neutral with regard to jurisdictional claims in published maps and institutional affiliations.

## Figures and Tables

**Table 1:** Physical and metabolic parameters of the animals.

BWT(g)					BG(mM)				UV(ml)			
Group	8 w	12 w	16 w	20 w	8 w	12 w	16 w	20 w	8 w	12 w	16 w	20 w
*db/m*	14.77 ± 1.80	16.94 ± 3.13	18.23 ± 1.32	20.06 ± 1.06	4.96 ± 0.75	5.06 ± 0.81	4.70 ± 1.21	5.0 ± 1.21	1.1 ± 0.9	1.5 ± 0.5	1.7 ± 0.8	1.2 ± 0.7
*db/m*+FA	15.90 ± 0.36	16.68 ± 2.89	17.86 ± 1.74	19.37 ± 0.98	5.33 ± 0.58	4.33 ± 1.23	5.60 ± 1.74	4.79 ± 1.06	1.3 ± 0.5	2.1 ± 0.4	1.1 ± 0.5	1.7 ± 0.6
*db/m*+NS	16.27 ± 0.47	18.26 ± 2.55	19.86 ± 1.32	20.12 ± 1.36	5.20 ± 0.52	5.06 ± 0.35	4.87 ± 0.59	5.28 ± 0.89	1.7 ± 0.5	1.2 ± 0.2	2.1 ± 0.5	1.5 ± 0.7
*db/db*	34.40 ± 1.95*	55.54 ± 1.69*	58.47 ± 1.26*	59.63 ± 2.27*	11.13 ± 1.77*	11.73 ± 1.66*	10.66 ± 1.59*	12.83 ± 2.76*	4.6 ± 1.0*	6.1 ± 1.5*	5.8 ± 1.6*	3.2 ± 1.3*
*db/db*+FA	32.70 ± 1.34*	52.74 ± 1.69*	56.56 ± 1.63*	60.34 ± 2.36*	11.33 ± 1.02*	10.82 ± 1.10*	11.73 ± 1.63*	12.73 ± 1.49*	5.4 ± 0.5*	5.5 ± 1.1*	4.6 ± 0.9*	4.8 ± 1.0*
*db/db*+NS	34.33 ± 1.95*	55.81 ± 2.97*	56.87 ± 1.54*	57.93 ± 1.38*	12.07 ± 0.20*	11.82 ± 1.02*	10.69 ± 1.47*	9.7 ± 1.0*	5.4 ± 0.5*	4.7 ± 0.8*	3.8 ± 1.2*	3.6 ± 0.6*

Abbreviations: BWT: body weight, BG: blood glucose, UV: urine volume, FA: Fasudil, NS: normal saline. ^*^P < 0.05 V.S. control. Abbreviations: db/db + NS, db/db+ normal saline. ^*^P < *0.05 V.S. control.*
